# Rapid Regulation of Alternative Splicing in Response to Environmental Stresses

**DOI:** 10.3389/fpls.2022.832177

**Published:** 2022-03-04

**Authors:** Xiao-Xiao Liu, Qian-Huan Guo, Wei-Bo Xu, Peng Liu, Kang Yan

**Affiliations:** ^1^State Key Laboratory of Crop Biology, College of Life Sciences, Shandong Agricultural University, Tai’an, China; ^2^Donald Danforth Plant Science Center, St. Louis, MO, United States

**Keywords:** alternative splicing, environmental stress, post-transcriptional regulation, splicing factor, epigenetic control

## Abstract

Plants overcome the changing environmental conditions through diverse strategies and complex regulations. In addition to direct regulation of gene transcription, alternative splicing (AS) also acts as a crucial regulatory mechanism to cope with various stresses. Generating from the same pre-mRNA, AS events allow rapid adjustment of the abundance and function of key stress-response components. Mounting evidence has indicated the close link between AS and plant stress response. However, the mechanisms on how environmental stresses trigger AS are far from understood. The advancing high-throughput sequencing technologies have been providing useful information, whereas genetic approaches have also yielded remarkable phenotypic evidence for AS control of stress responses. It is important to study how stresses trigger AS events for both fundamental science and applications. We review current understanding of stress-responsive AS in plants and discuss research challenges for the near future, including regulation of splicing factors, epigenetic modifications, the shared targets of splice isoforms, and the stress-adjusting ratios between splicing variants.

## Introduction

Since RNA splicing was initially discovered in 1977, this process of removing introns from pre-mRNA has been observed in most eukaryotic cells (Berget et al., 1977). The accuracy of RNA splicing is crucial for the synthesis of functional proteins. Although there are multiple splicing mechanisms, canonical splicing which is catalyzed by spliceosome accounts for the majority. The selection of splice sites is not only determined by core spliceosomal components but also regulated by a number of spliceosome-associated RNA binding factors, predominantly serine/arginine-rich (SR) proteins and other splicing factors (Laloum et al., 2018).

In response to changing environmental conditions, alternative mature transcripts from the same pre-mRNA can be generated rapidly by choosing different splicing sites (Laloum et al., 2018). AS greatly enhances the coding capacity of a genome and expands the proteome, regulating up to 95% of human and 70% of plant multi-exon genes (Pan et al., 2008; Zhang et al., 2010, 2017; Marquez et al., 2012). Intron retention (IR) is predominant in plants and exon skipping (ES) is the most frequent AS event in mammals (Gupta et al., 2004; Wang and Brendel, 2006). Notably, RNA-seq data have been confirming previous indications that abiotic stress markedly enhances AS events in plants (Laloum et al., 2018).

According to a recent study, different stresses rarely induce overlapped AS events in plants, suggesting an environmental specificity of AS regulations (Punzo et al., 2020). The selection of alternative splice sites was enhanced for more than 6,000 genes in *Arabidopsis* under high salinity (Feng et al., 2015). A large number of differential AS events in maize leaves were found when exposed to heat stress, and more than half of them are ES and IR (Li Z. et al., 2021). During the sharp cooling treatment on tea plants, the numbers of AS events were also significantly increased (Li et al., 2020). These observations have indicated that pre-mRNA splicing may have a strong bearing on stress response in plants. AS patterns can be altered directly by splicing factors or epigenetic changes, here we will review current studies on how stresses control AS through these mechanisms in plants. We will also discuss the consequences of AS and its influences on plant stress responses (Table 1).

**TABLE 1:** A summary of stress-responsive AS genes.

Gene	Specie	Stress responses	Function	Regulation types of AS*	References
*SR34b*	*Arabidopsis*	Cadmium	Splicing factors	A	Zhang et al., 2014
*SR45*	*Vitis vinifera*	Heat	Splicing factors	A	Jiang et al., 2017
*SR30*	*Vitis vinifera*	Heat	Splicing factors	A	Jiang et al., 2017
*SR34*	*Vitis vinifera*	Heat	Splicing factors	A	Jiang et al., 2017
*SCL*	*Arabidopsis*	ABA	Splicing factors	A	Cruz et al., 2014
*SR45a*	*maize*	Heat	Splicing factors	A	Li Z. et al., 2021
*STA1*	*Arabidopsis*	Cold	Splicing factor	A	Lee et al., 2006
*SKIP*	*Arabidopsis*	Salt	Splicing factor	A	Feng et al., 2015
*SME1*	*Arabidopsis*	Cold	Splicing factor Sm protein	A	Huertas et al., 2019
*RDM16*	*Arabidopsis*	ABA	ABA responsive splicing factor	A	Huang et al., 2013
*PRP18*	*maize*	Drought	Splicing factor	A	Thatcher et al., 2016
*GRP7*	*Arabidopsis*	Salt	Glycine-rich RNA binding protein	A	Wang et al., 2020
*SKB1*	*Arabidopsis*	Salt, ABA	Shk1 kinase binding protein	B	Zhang et al., 2011
*MET1-2*	*Rice*	Cadmium	CG methyltransferase	B	Wang et al., 2015
*FLC*	*Arabidopsis*	Salt	Transcription factor	B	Zhang et al., 2011
*HAB1*	*Arabidopsis*	ABA	Type 2C protein phosphatase	C D	Wang et al., 2015
*CIPK3*	*Arabidopsis*	ABA, Drought	Serine-threonine protein kinase	C	Sanyal et al., 2017
*HsfA2*	*Arabidopsis*	Heat	Transcription factor	C	Liu et al., 2013
*HSFA*	*Tomato, Lily*	Heat	Transcription factor	C	Hu Y. et al., 2019; Wu et al., 2019
*IDD14*	*Arabidopsis*	Cold	Inderminate domain	C	Seo et al., 2011
*SR45a*	*Arabidopsis*	Salt	SR like protein	C D	Li Y. et al., 2021
*SRAS1*	*Arabidopsis*	Salt	Salt-responsive AS gene	C D	Zhou et al., 2021
*CBP20*	*Arabidopsis*	Salt	Cap-binding protein	C	Li Y. et al., 2021
*CSN5A*	*Arabidopsis*	ABA, Salt	COP9 signalosome	C	Zhou et al., 2021
*ABI3*	*Arabidopsis*	ABA	Transcription factor	D	Sugliani et al., 2010
*RBM25*	*Arabidopsis*	ABA	RNA-binding protein	D	Cheng et al., 2017
*VvPMA1*	*Vitis vinifera*	Salt	PM H^+^-ATPase genes	D	Han et al., 2017
*LUC7*	*Arabidopsis*	Cold	Lethal unless CBC7	D	Marcella et al., 2018
*FLM*	*Arabidopsis*	Heat	MADS domain protein	D	Chang et al., 2021
*CML21*	*Vitis vinifera*	Cold	Calmodulin-Like Gene	D	Aleynova et al., 2020

**FIGURE 1:**
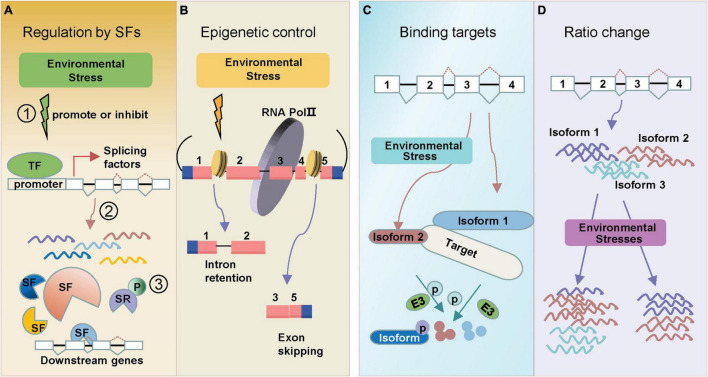
Regulation types of AS in response to environmental stresses. Alternative splicing under environmental stresses. **(A)** Stresses can induce alternative splicing through splicing factors (SF). SFs are regulated at multiple levels: (1) environmental stress promotes or inhibits transcription of splicing factors; (2) many splicing factors themselves generate alternative splicing events which further regulate splicing of downstream genes; (3) SR proteins can be phosphorylated (labeled “P”) under stress. **(B)** Environmental stresses change epigenetic marks which alter the pol II elongation rate, leading to either Intron retention (IR) or Exon skipping (ES). **(C)** Environmental stress induces different splicing isoform (isoform 2), which tends to bind the same target (protein or DNA) that full-length isoform (isoform 1) binds. One example is the splice isoforms involved in the regulation of protein degradation. **(D)** Alternative splicing generates transcript isoforms with variable abundance. Environmental stresses lead to the altered ratios of splice variants. Exons are displayed as boxes and introns as lines in gene diagrams.

*Different types of AS are illustrated in the Figure 1.

## Direct Regulation by Splicing Factors

Alternative splicing is most commonly controlled by splicing factors, and AS regulation studies have been primarily focusing on key RNA sequence elements and their associated regulators (Luco et al., 2011). Plant stress-responsive genes are particularly prone to generating multiple transcripts in response to different environmental stresses (Ner-Gaon et al., 2004). However, the conserved and specific stress-responsive RNA sequence elements were not found among plant species, and AS events from different genes revealed completely diversified splicing recognition sites (Laloum et al., 2018). These suggest that the splicing mechanism in response to environmental stresses is more dependent on a variety of splicing factors in plants.

Splicing factor genes usually show a quick response to environmental stresses at transcriptional levels (Laloum et al., 2018). For example, the SR34b gene is upregulated by cadmium (Cd), and controls the plant tolerance to Cd toxicity in *Arabidopsis* (Zhang et al., 2014). Two plant-specific SC35-Like (SCL) SR genes are downregulated upon exposure to exogenous ABA treatment in *Arabidopsis* (Cruz et al., 2014). The U5 snRNP–associated splicing factor, STABILIZED1 (STA1) gene is upregulated in response to cold stress, and the phenotypes of *sta1-1* plants under cold stress is severe (Lee et al., 2006). The transcriptional and protein levels of the Ser/Arg-rich splicing factor SR45, SR30, and SR34, and the nuclear ribonucleic protein U1A accumulate under high temperature in *Vitis vinifera* (Jiang et al., 2017). Also, the expression levels of splicing factor PRP18 shows large increases under drought conditions in maize (Thatcher et al., 2016). Some splicing factors themselves undergo AS events under stress, like SR45a, in which two splice isoforms showed strong induction by salt treatment (Li Y. et al., 2021).

The change of splicing factor transcriptional expressions usually leads to the altered AS patterns of downstream stress-responsive genes (Figure 1A). Ski-interacting protein (SKIP) is a salt-responsive splicing factor, which mediates the AS of many genes in the recognition and cleavage of 5′ donor and 3′ acceptor sites (Feng et al., 2015). Salt stress changes SKIP expression levels and decreases the ability of the spliceosome to accurately recognize splice sites (Feng et al., 2015). Splicing factor Sm protein E1 (SME1) ensures the appropriate splicing of a high number of pre-mRNAs in maintaining the levels of selected cold-responsive functional transcripts (Huertas et al., 2019). *SME1* shows a cold response expression pattern, and the mutant phenotype displays that SME1 functions as a negative regulator of the cold acclimation process by regulating splicing events (Huertas et al., 2019). RNA-directed DNA methylation 16 (*RDM16)* encodes an ABA responsive pre-mRNA-splicing factor 3(PRP3), which is involved in pre-mRNA splicing. And RNA-seq data identified 308 IR events changed in *rdm16* mutant (Huang et al., 2013).

In addition to the transcriptional regulations, splicing factors are also modified at post-translational levels. Most of the nuclear SR proteins are phosphorylated on their RS domain, and the phosphorylation status of SR proteins is highly related to their functions in spliceosome assembly and subcellular localization (Jeong, 2017). The phosphorylation of glycine-rich RNA binding protein7 (GRP7) enhances its mRNA binding ability and its association with spliceosome component U1-70K to change dynamic AS (Wang et al., 2020). The alkalinization of FACTOR 1 (RALF1) and FERONIA (FER) triggers rapid and massive AS events by interacting with and phosphorylating GRP7 in *Arabidopsis* (Wang et al., 2020).

## Epigenetic Control

Epigenetic markers, like chromatin modification and DNA methylation have also been found to be associated with AS regulations. Dramatic epigenetic changes play key roles in cell- and condition-dependent AS regulation in animals (Ibtissam et al., 2019). In plants, the role of epigenetic control in regulating AS under stress is emerging (Hu J. et al., 2019). Recent data have identified a strong relationship between chromatin changes and AS control. A potential epigenetic control of AS is through pol II (Figure 1B). The pol II initiation and elongation speed mediate the splicing processing of pre-mRNAs to generate AS transcripts in plants (Ibtissam et al., 2019). Greater pol II processivity is associated with a more open chromatin structure, which favors pol II elongation (Petrillo et al., 2014; Godoy Herz et al., 2019). In *Arabidopsis* and rice, the chromatin structure was more open in retained introns (Fahad et al., 2018). The open chromatin architecture enhanced pol II elongation rate, which led to skipping of splice sites (Fahad et al., 2018). In addition, pol II elongation speed was also found to be slower in exons than introns in *Arabidopsis* (Ibtissam et al., 2019). Interestingly, pol II elongation speed is faster under light conditions than in darkness, leading to an ES (Godoy Herz et al., 2019). DNA methylation is also associated with chromatin remodeling to regulate plant AS patterns. In rice, the widespread differences of splicing variants were found in CG methyltransferase mutant *OsMet1-2* lines (Wang et al., 2016). Also, CG methylation was higher in AS-related introns than constitutive introns (Wang et al., 2018).

A close relationship between abundant epigenetic modifications and splicing variation has been revealed under different growth and stress conditions. Under salt stress, PRMT5 (protein arginine methyltransferase 5) methyltransferase (also known as SKB1) increases H4R3sme2 (histone 4 arginine 3 symmetric demethylation) levels in *Arabidopsis*, suggesting SKB1 disassociation from chromatin results in a reduction in the cellular levels of H4R3sme2, resulting in the induction of FLOWERING LOCUS C (FLC) and salt stress-responsive genes (Zhang et al., 2011). PRMT5 also alters AS in the core clock gene PSEUDO RESPONSE REGULATOR 9 (PRR9) and influences clock functioning in *Arabidopsis* (Sanchez et al., 2010). Evidence in rice indicates that histone H3K36-specific methyltransferase (SDG725) regulates IR events in many genes (Wei et al., 2018). In *Arabidopsis*, temperature-induced differentially spliced genes are enriched in H3K36me3 marks, while depletion of H3k36me3 marks has the opposite effect to temperature-induced AS (Pajoro et al., 2017).

## Shared Target Binding and Regulating

In consequence of AS, novel protein products may be generated and involved in the plant stress responses. AS events often introduce premature stop codons, generating truncated isoforms. Interestingly, these truncated isoforms often keep the ability of interacting with the same target with full-length protein, while some ability is lost because of missing key domain (Figure 1C). In the ABA signaling pathway, the Group A protein type 2C phosphatases (PP2C) *HAB1* undergoes ABA-controlled AS to produce two splice variants, which encode HAB1.1 and HAB1.2. Both of them interact with subclass III SNF1-related protein kinases SnRK2.6 (OST1) in both cytoplasm and nucleus (Wang et al., 2015). Another example is the CIPK3 splice variants induced by ABA and drought treatment. Five CIPK3 protein variants (CIPK3.1, CIPK3.2, CIPK3.3, CIPK3.4, and CIPK3.5) have been generated. Although having different preferences on their upstream CBL interactors, these proteins do not lose the binding ability to their target (Sanyal et al., 2017).

A key transcription factor Heat shock transcription factorA2 (HsfA2) generate a new intron-retained splice variant (designated *HsfA2-III*) in *Arabidopsis* seedlings (Liu et al., 2013). This truncated isoform still has DNA-binding ability, but the loss of C-terminal activation domain (CTAD) of HsfA2 might lead to the defect of transactivation activity and failure in functioning as a transcription factor (Liu et al., 2013). Similar AS events of HSFA also occur in *Tomato* and *Lily* (Hu Y. et al., 2019; Wu et al., 2019). The generation of HSFA splice variants might result in a genetic buffering to tolerate the negative effects of long-term Heat stress on plants (Hu J. et al., 2019). Another temperature-dependent case is the INDERMINATE DOMAIN 14 (IDD14). An alternatively spliced IDD14 form (IDD14β), which is generated under cold conditions, lacks functional DNA binding domain but is able to form heterodimers with the functional IDD14 form (IDD14α) (Seo et al., 2011).

Two splicing variants of serine/arginine-rich (SR)-like protein (SR45a) were also identified under salt stress, full-length SR45a-1a and the truncated isoform SR45a-1b. The full-length SR45a-1a works as a splicing factor while the truncated isoform SR45a-1b does not (Li Y. et al., 2021). However, the cap-binding complex subunit cap-binding protein 20 (CBP20) indeed physically interacts with both SR45a-1a and SR45a-1b. SR45a-1b mediates salt-stress signal transduction pathways through promoting the association of SR45a-1a with CBP20 (Li Y. et al., 2021). Another example is the *Salt-Responsive Alternatively Spliced gene 1* (*SRAS1*), encoding a RING-Type E3 ligase. It can generate two splicing variants: *SRAS1.1* and *SRAS1.2*, which exhibit opposing responses to salt stress (Zhou et al., 2021). The full-length SRAS1.1 targets and promotes the degradation of CSN5A, while SRAS1.2 protects CSN5A by competing with SRAS1.1 on the same binding site (Zhou et al., 2021).

## Adjustment of Splicing Variant Ratios

In response to environmental changes, new splicing variants might emerge from stress-responsive genes (Laloum et al., 2018). Simultaneously, the expression levels of original splicing variants might also alter, resulting in an adjustment of splicing variant ratios (Figure 1D). These changes may be essential for plants to adapt to environmental challenges. Following up the above discussion on HAB1, expression analysis showed that the HAB1.2/HAB1.1 ratio greatly differed at the germination stage with or without ABA treatment (Wang et al., 2015). Under drought treatment, the ratios of HAB1.2/HAB1.1 increased at 0.5 h and then decreased as time went on in the WT (Cheng et al., 2017). RNA-binding protein 25 (RBM25) acts upstream of HAB1, a conserved ABA-induced splicing factor, affecting the ratio of HAB1.2/HAB1.1 to modulate plant response to drought stress (Cheng et al., 2017). There are also some other ratio shifts of splicing variants responding to ABA. Splicing factors suppressor of ABI3 (SUA) suppresses splicing of the cryptic *ABSCISIC ACID INSENSITIVE3 (ABI3)* intron and thereby influences the ratio between ABI3-α and ABI3-β transcripts, which then could represent a system to finetune seed maturation (Sugliani et al., 2010).

Similar phenomena were also observed under salt stress. Salt stress triggered the ratio changing of SR45a (SR45a-1a and SR45a-1b) and SRAS1 (SRAS1.1 and SRAS1.2) (Li Y. et al., 2021; Zhou et al., 2021). Another interesting example is the AS events of *Vitis vinifera* PM H^+^-ATPase genes 1 (VvPMA1). The ratio changing of VvPMA1 was discovered under salt conditions (Han et al., 2017). Subtle changes in the ratio of VvPMA1α and VvPMA1β likely have profound effects on PM H^+^-ATPase activity in grape root under salinity (Han et al., 2017).

Besides ABA and salt, extreme temperature also has a dramatic effect on the splice patterns of many genes. Heat and cold stress alter the ratios of splice isoforms for many SR family members, such as *SR34b*, RS41, and *SR30* (Palusa et al., 2007). One additional splice form (isoform 9) of *SR34b* appeared in heat-treated seedlings, whereas some isoforms (3, 6, 7, and 8) were transcriptionally reduced. The ratios of *SR34b* transcripts altered in seedlings with heat or cold treatment (Palusa et al., 2007). The levels of all four splice variants of the Grapevine Calmodulin-Like Gene (VaCML21) were highly induced in response to cold stress (Aleynova et al., 2020). The ratios between transcript variants are changing with or without cold stress (Aleynova et al., 2020). LETHAL UNLESS CBC7 (LUC7) proteins specifically promote a subset of terminal introns splicing in response to cold stress, leading to the ratio changing of splicing variants of cold responsive gene (Marcella et al., 2018).

In *Arabidopsis*, FLOWERING LOCUS M (FLM) undergoes AS and this temperature-dependent AS leads to a differential accumulation of the FLM-β and FLM-δ transcripts (Lee et al., 2013). Two main splicing variants compete for interaction with the floral repressor SVP to control temperature-dependent flowering (Posé et al., 2013). FLM-β was the prevalent splice variant at 16 °C, whereas FLM-δ dominated at 27 °C. The splice variant ratios of FLM are regulated in response to low and high temperature coupled with nonsense-mediated mRNA decay pathway (Sureshkumar et al., 2016). Also, the splicing events of FLM are mediated by another splicing factor 1 (AtSF1) during its signaling pathway (Kim et al., 2020). AtSF1 acting in 3′ splice-site recognition is responsible for ambient temperature-dependent AS of FLM pre-mRNA, resulting in the temperature-dependent production of functional FLM-β transcripts (Kim et al., 2020). The cyclin-dependent kinase G2 (CDKG2), together with its cognate cyclin, CYCLYN L1 (CYCL1) affects the AS of FLM, balancing the levels of FLM-β and FLM-δ across the ambient temperature range (Nibau et al., 2020). Both the level and splicing pattern of FLM transcripts are affected by RBP45d and PRP39a, which facilitate temperature-induced AS of FLM to induce flowering at higher temperature (Chang et al., 2021).

## Conclusion and Future Prospects

Evidence has indicated that AS, acting as a crucial regulatory mechanism in response to various stresses, is fast and efficient, and this may have an evolutionary advantage for plants to survive under rapidly changing environments. Although splice variants participate in different pathways, they do have something in common: (1) alternatively spliced transcripts tend to encode truncated proteins that interact with the same targets; and (2) the ratios of splice variants are critical in the regulation of stress response pathways. A key question is how stress signals control splicing factors and lead to AS? Previous studies have shed some light. The reversible phosphorylation might be crucial in the regulation of splicing factor activity. Epigenetics is also associated with AS, and histone markers can alter the pol II elongation speed leading to either ES or IR. More in-depth studies will be necessary to address the upstream regulatory pathways of AS. Another question is how stress-induced AS helps plants to adapt to environmental challenges. More case-by-case studies will be needed to better understand the whole picture.

In addition, CRISPR/Cas9 technology has given us power to engineer the splicing patterns of trait genes for improving crops. The identification of splice variants and function characterization are very helpful for developing new gene editing approaches. Take the FLM variants for example, native FLM genomic locus can be directly edited by deleting exon 2 or 3 to generate splice variants, and the generated germlines display different flowering phenotypes (Capovilla et al., 2017). Instead of deleting entire exons, we could directly modify splice sites in crop genomes to generate desired transcript variants using CRISPR/Cas9 based base editor. The CRISPR/dCas9 could also be coupled with methylation/demethylation enzymes to modulate splicing outcomes through mediating pol II speed. Future studies on stress triggered AS will be of great value in understating the mechanisms of regulation of AS, and in improving crop adaptations to extreme environmental conditions.

## Author Contributions

All authors listed have made a substantial, direct, and intellectual contribution to the work, and approved it for publication.

## Conflict of Interest

The authors declare that the research was conducted in the absence of any commercial or financial relationships that could be construed as a potential conflict of interest.

## Publisher’s Note

All claims expressed in this article are solely those of the authors and do not necessarily represent those of their affiliated organizations, or those of the publisher, the editors and the reviewers. Any product that may be evaluated in this article, or claim that may be made by its manufacturer, is not guaranteed or endorsed by the publisher.
